# Surfing Corona waves – instead of breaking them: Rethinking the role of natural immunity in COVID-19 policy

**DOI:** 10.12688/f1000research.110593.3

**Published:** 2023-08-24

**Authors:** Andreas Kalk, Joachim Sturmberg, Wim Van Damme, Garrett W. Brown, Valéry Ridde, Martin Zizi, Elisabeth Paul

**Affiliations:** 1 Kinshasa Country Office, Deutsche Gesellschaft für Internationale Zusammenarbeit (GIZ), Kinshasa, Democratic Republic of the Congo; 2 Foundation President – International Society for Systems and Complexity Sciences for Health, Australia, Callaghan, Australia; 3 A/Prof of General Practice, College of Health, Medicine and Wellbeing, University of Newcastle, Australia, Callaghan, Australia; 4 Department of Public Health, Institute of Tropical Medicine, Antwerp, Antwerp, Belgium; 5 Global Health Theme, University of Leeds, Leeds, UK; 6 CEPED, IRD-Université de Paris, ERL INSERM SAGESUD, Institute for Research on Sustainable Development (IRD), Paris, France; 7 Aerendir Mobile Inc., Mountain View, California, USA; 8 School of Public Health, Université libre de Bruxelles, Brussels, 1070, Belgium

**Keywords:** COVID-19; SARS-CoV-2; vaccines; natural immunity; health policy

## Abstract

In the first two years of the pandemic, COVID-19 response policies have aimed to break Corona waves through non-pharmaceutical interventions and mass vaccination. However, for long-term strategies to be effective and efficient, and to avoid massive disruption and social harms, it is crucial to introduce the role of natural immunity in our thinking about COVID-19 (or future “Disease-X”) control and prevention. We argue that any Corona or similar virus control policy must appropriately balance five key elements simultaneously: balancing the various fundamental interests of the nation, as well as the various interventions within the health sector; tailoring the prevention measures and treatments to individual needs; limiting social interaction restrictions; and balancing the role of vaccinations against the role of naturally induced immunity. Given the high infectivity of SARS-CoV-2 and its differential impact on population segments, we examine this last element in more detail and argue that an important aspect of ‘living with the virus’ will be to better understand the role of naturally induced immunity in our overall COVID-19 policy response. In our eyes, a policy approach that factors natural immunity should be considered for persons without major comorbidities and those having ‘encountered’ the antigen in the past.

## Introduction

### Coronavirus control policies, a short history

The COVID-19 pandemic caused by a novel coronavirus, SARS-CoV-2, emerged late in 2019 in Wuhan, China, and spread within a few months around the globe, causing epidemics of variable intensity in diverse contexts. ^1^ Genetic mutations created thousands of new variants of the virus, some of them being more infectious than the original variant and becoming principal drivers of the pandemic, recently the Delta variant, superseded by the Omicron variant, are so far the most infectious (but less virulent?). ^2^ At the time of writing this paper (September 2022), over 620 million COVID-19 cases and more than six and a half million related deaths have been registered around the world. ^3^

The initial policy response to the pandemic in most countries was characterised by two factors. On the one hand, very few governments explicitly defined an overall policy goal for coronavirus control. Implicitly, some governments (like China) seemed to strive for elimination of the epidemic (eradication of a zoonosis being next to impossible), others (like Brazil or the USA) depicted it ‘like a bad flu’ and tried – at least for a given period – to basically ignore it. ^4^ On the other hand, activities to control the pandemic showed an increasing degree of conformity across countries, the main pillars being initially ‘non-pharmaceutical interventions’ (NPIs) including lockdown measures, travel restrictions and face mask mandates, but soon, incorporated mass vaccination of the population in order ‘to break the waves’ (peaks of the epidemic in terms of hospitalisations and deaths, the main acknowledged policy objectives in most countries).

Simultaneously, and in the absence of an explicit overall policy goal, secondary objectives such as ‘flattening the curve’ and ‘protecting health services from being overwhelmed’ were intensively discussed and imposed. The logic was simple, but also a gamble – use NPIs to slow the virus while waiting for vaccine discovery, rollout and mass inoculation. ^5^ Yet, the need for protecting services also reflected prior negligence, where austerity policies, disinvestment in primary healthcare, and poor preventative and preparedness measures left systems struggling to cope, while also widening pre-existing inequities. These further policy developments went hand in hand with the slow recognition that it was neither feasible to eliminate the disease nor to continuously ignore it. And they slowly coincided with an emerging understanding that vaccines alone cannot offer the miracle solution many governments had hoped for decreasing the number of infections or ‘cases’. Although most countries continue to act without a clear definition of an overarching policy goal – instead re-imposing knee-jerk NPI policies to reduce risk – there is now seemingly increased recognition that humankind will have to ‘to live with the virus’ one way or another. ^6^ This recognition is crucial, since SARS-CoV-2, like all coronaviruses, is a zoonosis, having numerous mammalian host species. ^7^^–^^10^ Not all those hosts represent a principal reservoir of the virus, but species can *de facto* ping-pong mutations between them, enhancing genetic variations and thus jeopardising virus control.

What remains unclear and highly elusive are articulated long-term strategies for ‘living with the virus’, which can be effective and efficient, without causing the massive disruption and social harms associated with current coronavirus policies. Toward that end, it is crucial to consider basic immune system behaviour, and secondly, to better factor the known specific immunological pathways of SARS-CoV-2. In this paper, we propose to introduce an additional policy component in response, namely, the role of natural immunity – stimulating polyclonal antibody (B-cell) and cellular (T-cell) responses – in our thinking about COVID-19 control and prevention. We therefore do not criticise the resort to NPIs or vaccination campaigns at the beginning of the pandemic, but question their value after several pandemic waves when a certain degree of immunity had been attained worldwide. However, our starting point prior to the presentation of this argument is to suggest that any coronavirus – or similar virus causing “Disease X” in the future – control policy (including this our alternative offered here) must appropriately balance five key elements simultaneously. Our rationale is suitable for high-, middle- and low-income countries, with an appropriate balance to be found for each context, according to local epidemiological characteristics, health system capacities and values.

### The art to balance, five considerations

***First, the interest to protect the population from Covid-19 has to be appropriately balanced against other fundamental interests of the nation***

These interests include respect for personal liberty and human rights on one side, and the need to protect local and the global economies on the other. For example, the former aspect requires serious consideration of the right to education, since school closures are one of the least evidence-based NPIs ^11^^–^^14^ and there is credible evidence that children and adolescents are far less susceptible to illness and serious disease. ^15^ The latter (economic) aspect has to include often forgotten informal economies, since in many countries (particularly low- and middle-income countries) they represent a major source of economic activity and livelihood. Thus, policy making must better consider the economic effects both of the pandemic as well as the control measures. This is particularly germane since there is growing evidence demonstrating the adverse long-term economic and health effects of many NPIs. Finding an appropriate balance between these elements is important since education and socio-economic development are crucial determinants of health in the medium term, ^16^ while producing epigenetic changes for several generations to come. ^17^ The respect of such a balance of coronavirus control measures against other fundamental interests can be understood as an essential element of the social contract between government and citizens, contributing significantly to trust in government authority and bonds of social solidarity.

As pointed out by Kass ^18^ and later by Turcotte-Tremblay and Ridde, ^19^ finding an appropriate balance between health, liberty and other social concerns should review the available scientific evidence in light of ethical frameworks that can better reflect concerns for equity and social justice, thus incorporating wider considerations than those provided merely through morbidity and mortality calculations. Such a process is not easy and requires consultative and deliberative processes that can reflect multifarious interests, population strata, and sectors, rather than relying on the view of a limited cohort of specialists ^20^ – which often promotes ‘groupthink’, underrepresentation, and ‘enclave echo chambers’. ^21^

***Second, coronavirus control has to be balanced against other interventions within the health sector***

This challenge again entails three aspects: The allocation of human, financial and physical resources must correspond to the relative burden of disease in a given community. ^22^ As of now, in many countries (e. g. sub-Saharan Africa), other health threats such as neonatal disorders, lower respiratory infections, malaria, and tuberculosis cause a significantly higher burden of disease than COVID-19. ^23^ Of equal importance is the consideration of the negative health-related effects of coronavirus control ranging from psychological damage, particularly from the promulgation of fear, the interference to supply chains resulting in malnutrition (again especially in LMICs), and the deterioration of other pre-existing diseases (and even death) caused by travel restrictions, health system lockdowns, and other measures. Thirdly, health interventions must be evaluated in terms of all their effects on health and health systems: for instance, Covid-19 vaccines may prevent some Covid-19 related hospitalisations, but also incur costs in terms of health system resources at the time of vaccination ^24^ as well as for caring for additional adverse event. ^25^

***Third, preventive measures have to be balanced against the need to assure and improve – and tailor to individual needs – the treatment of patients affected by COVID-19***

We insist that pharmaceutical interventions represent one of the four cornerstones of coronavirus control (the others being preventive: NPIs, vaccination and natural immunity). It is unprecedented how quickly researchers identified, over the past months, drugs to treat the disease. Some of them were known, sometimes even for similar indications (as dexamethasone, ^26^ heparin – preventively ^27^ and/or therapeutically ^28^ – or xylitol nasal spray as a preventative); ^29^ some represent newer developments (antivirals as molnupiravir ^30^ or monoclonal antibodies). ^31^ Yet, these advances have so far been largely overshadowed by a policy focus obsessed with the use of NPIs and vaccines.

***Fourth, within the toolbox of preventive measures, most NPIs rely on reduced contact between human beings which runs counter to our social nature***

Large scale and blanket NPIs have been recommended based on models that did not take into account the fact that SARS-CoV-2, like all coronaviruses, can have numerous interspecific hosts. ^10^ Moreover, the evidence for the efficacy of ‘NPIs in practice’ is lacking, ^32^ while evidence for their negative consequences has been consistently growing. ^33^ Most NPIs provoke social segregation and must thus be seen as being in tension with our social nature while amplifying emotional distress. Catastrophic consequences of psychological damage (particularly for frontline healthcare workers, over and above adverse physical events, ^34^ the elderly, children and people working in the informal care sector) have been observed on all continents. ^35^^–^^38^ Consequently, priority should be given to prevent COVID-19 for those at greatest physical and/or emotional risk by achieving immunity, be it induced ‘naturally’ or ‘artificially’ through vaccination. Moreover, priority should be given to NPIs which do not incur adverse events on health and well-being, such as aeration and filtration of indoor places. ^39^

***Lastly, the role vaccinations should play now, and in the future of coronavirus control, has to be balanced against the role naturally induced immunity already plays and could play in the future***

Given the high infectivity of SARS-CoV-2 and its differential impact on population segments, this particular question deserves greater attention. As a result, we examine this in more detail below, arguing that an important aspect of ‘living with the virus’ will be to better understand the role of naturally induced immunity in our overall COVID-19 policy response.

### Immunity against SARS-CoV-2 – the five-step ladder of immunological specificity

**Figure 1.**
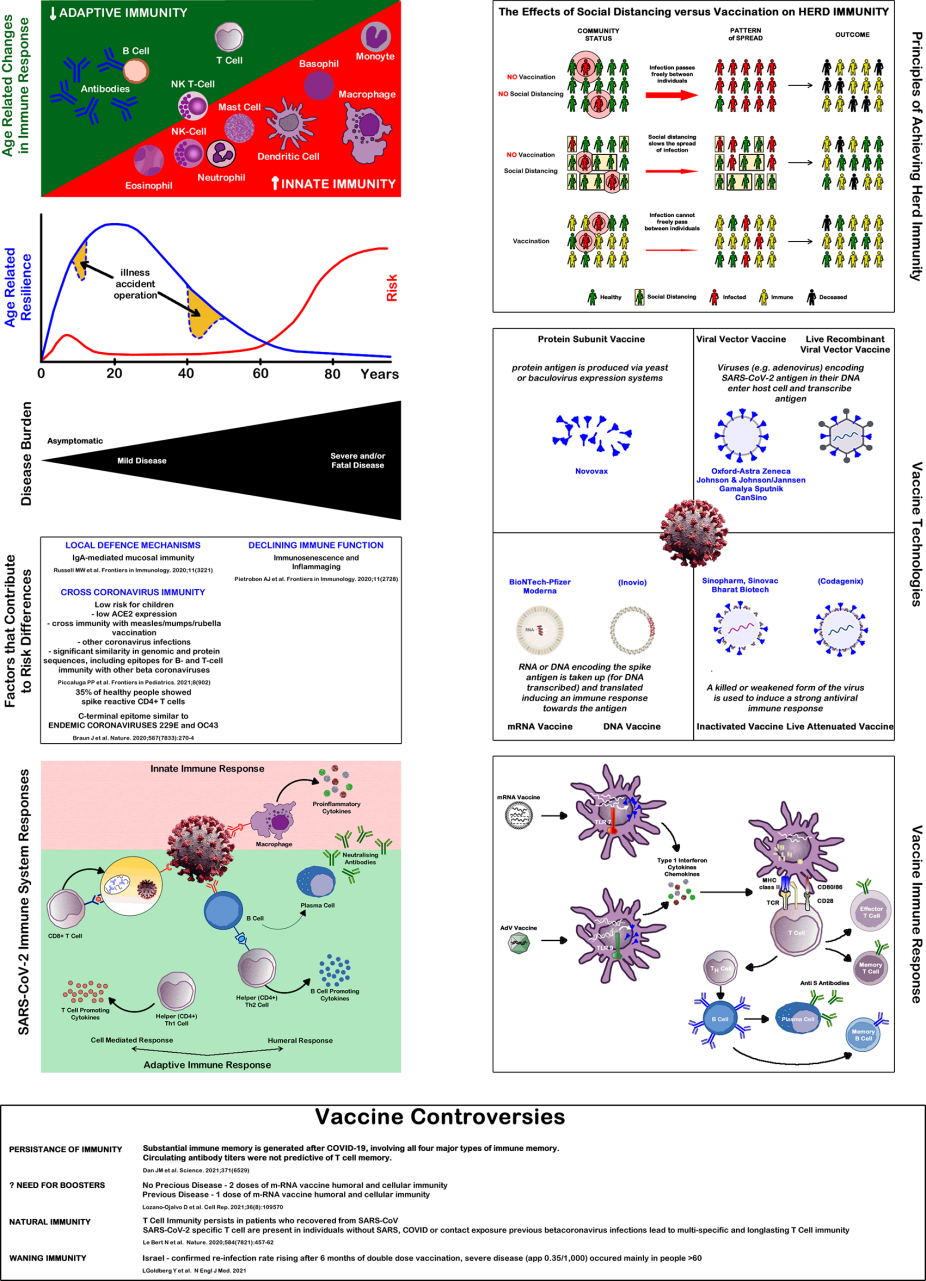
Immune system and Covid-19. Source: authors.

Innate immunity, which is the first line of defence of the immune system, is key to combat a novel virus such as SARS-CoV-2. ^40^ When analysing the factors inducing immunity against SARS-CoV-2, five different steps of increasing immunological specificity can be distinguished. These levels might be based on different immunological mechanisms and pathways, which are outlined below and summarized in Figure 1.

***First***, most people have some kind of natural resistance against pathogens ^41^ or ‘general immune competence’ and are thus distinguished from those with known immune deficiency diseases or immune system compromise. The macrophages are one of the responsible actors. ‘Generally immune competent’ individuals are less prone to become infected or to become sick once infected. Such competence (based partially on a rapid interferon response) might explain the fact that children are much less affected by COVID-19 than adults. ^42^^,^^43^

***Second***, one’s immune system is likely to be ‘trained’ in the course of one’s life through a large variety of infections without a higher degree of genetic similarity among the infectious agents. Thus, previous helminthic infections as well as malaria are likely to protect gradually from other infections, most notably by SARS-CoV-2, and from more serious forms of COVID-19. It is possible that the slow progress of the coronavirus pandemic in some countries can partially be explained by this phenomenon. ^44^

***Third***, it is known since 1970 that coronaviruses provoke 10-20% of all ‘flu waves’ and ‘common colds’ experienced globally. Previous exposure to such ‘older’ coronaviruses most likely fosters some level of cross immunity against SARS-CoV-2 and its different variants. ^45^^,^^46^

***Fourth***, a previous infection with SARS-CoV-2 creates a certain degree of immunity against reinfection. This immunity can result both from symptomatic as well as from asymptomatic infections, the latter possibly characterised by a higher degree of immunity, notably because the virus is polyclonal and not limited to the highly mutating spike protein, and because it fosters stronger mucosal and T-cell responses. ^47^^–^^56^

***Fifth***, immunity against the virus can be induced by vaccines. Whilst the aforementioned forms of immunity are caused ‘naturally’, vaccine-induced immunity represents an artificially created defence mechanism, generally based only on parts of the virus, principally the spike protein (viral vector and m-RNA vaccines), and inactivated or attenuated vaccines. Available evidence is not yet conclusive and partially contradictory, but vaccine-induced immunity might be more susceptible to evasion by new virus variants. ^57^^–^^60^ Moreover, bivalent vaccines do not appear to have a strong incremental capacity to reduced infections, compared to original vaccines. ^61^

Most countries, both in high-income and low- and middle-income settings, have now experienced several waves of coronavirus infections, and with this evolving experience, the strategies should be adapted. If one agrees that the elimination of SARS-CoV-2 is untenable, and if one accepts that the continued use of NPIs is not sustainable over the long run, then, by deduction, the best epidemic exit strategy would need to be based on the two remaining preventive pillars, namely, ‘natural’ immunity and vaccination. In this context, it is highly surprising that most governmental policies still rely largely on vaccination and ignore the role other forms of immunity could play and do play already. This is particularly astonishing now that the evidence strongly suggests that the efficacy of vaccinations wanes quite rapidly. ^62^^–^^66^ Indeed, Goldberg et al. have shown that natural immunity is more effective in preventing confirmed reinfection than two doses of vaccine, and that hybrid immunity of infection and a single vaccine dose provides the best protection. How long any form of immunity ultimately lasts remains unclear, and so far, there are no data to show the longer-term benefits of three vaccine doses. COVID-19 hospitalisations appear to occur at the same rate for people with natural or vaccine induced immunity. ^67^

In addition, the arrival of the Omicron variant and the newly emergent variants (BA.4 and BA.5) powerfully illustrates that such vaccine efficacy will be constantly challenged by mutations. ^68^^,^^69^ However, new mutations seem also increasingly “to run out of steam” with the rising level of community immunity. ^69^ While these two latest variants spread faster, they also cause less disease and hospitalisations. SARS-CoV-2, regardless of its subtype, is reaching an endemic state comparable to other common viruses ^69^ – like in the case of traditional influenza vaccines, but unlike the efficacy of vaccines against ‘stable’ viruses as mumps, measles or yellow fever. ^70^^,^^71^ Omicron has also demonstrated that current policies remain disjointed according to national self-interest, that good science and reporting is not always rewarded (as in the case of South Africa reporting Omicron), and that outdated notions of national health security (particularly in the West) will only be as strong as their global weakest links.

As far as the role of natural immunity is concerned, it must be recognised that people having had a proven SARS-CoV-2 infection (as long as confirmed by a previous positive PCR/antigen test) are registered in many countries, and they enjoy privileges similar to those with a complete vaccination record (in terms of movement restrictions, access to public places etc.) – at least for a few months. Nonetheless, so far, no effort has been made to identify systematically those people having either contracted COVID-19 or an asymptomatic/undiagnosed infection. Following data from the Institute for Health Metrics and Evaluation (IHME), this proportion is more than 50% in most countries in Africa and South Americas, and surpasses 85% in countries such as Algeria, Bolivia, Iran, Kenya, Russia and South Africa. ^72^

We defend the thesis that these figures have to be taken into account when promoting COVID-19 prevention through individual and collective immunity. Furthermore, the immunostimulant synergy between previous vaccinations and previous infections has to be further scrutinised beyond the only available study by Goldberg et al. ^67^ and reflected upon within public health policies.

The importance of conducting such an analysis is underpinned by several empirical conditions: i) the aforementioned high degree of peoples’ natural exposure to the virus and presumed widespread natural immunity in many countries, ii) the recognition that vaccine-induced protection wanes quite rapidly (in the course of 3-8 months), ^62^^,^^63^^,^^65^^,^^67^ and in consequence iii) the ever-increasing frequency of test-identified ‘breakthrough’ infections (infections of correctly vaccinated persons) presenting with limited or unspecific upper respiratory symptoms.

Hence, crucial questions arise: Do we really need anti-coronavirus ‘refresher jabs’ with the frequency promoted by many policy makers, media outlets and vaccine producers? Or should these ‘jabs’ be reserved for highly vulnerable people? Following such a booster strategy for the entire population is costly, intrusive, and hugely ignorant of the fact that current vaccine distribution has been inequitable, ^73^ with many parts of the world yet unable to access vaccines to protect at least those most vulnerable people. ^24^ It is hard to imagine how this will not continue under any booster reliant regime as it will operate within the already existing ‘vaccine apartheid (or inequity)’. ^74^

## Conclusion: a plea for a ‘mix’ of immune inductions by antigen exposure

Antia and Halloran distinguish three interrelated forms of immunity, which can all be caused both naturally and by vaccination: ^75^

• Immunity against infection;
• Immunity allowing infection, but reducing infectiousness;
• Immunity against disease (reducing disease manifestation and preventing death).

They stipulate – and this presumption is at least plausible – that immunity against infection wanes more rapidly than immunity against disease, the latter one being a weaker, but more important form of immunity mediated by T-cells. ^55^ This results in a time window labelled by Antia and Halloran as a ‘region of mild boosting’, namely, a period allowing re-infection or re-vaccination without developing (serious) disease. This window is depicted as most appropriate for refreshing the immunity to previous or even new levels. It might be open for a period ranging from approximately 3 to 24 months since the last antigen exposure, be it based on natural exposure or on vaccination. Moreover, Antia and Halloran insist that *‘… we also need to determine if multiple infections or vaccinations are needed to generate long-lasting protection against pathology, and whether this depends on the age of the individual’.*

We propose that the role of pre-existing or induced natural immunity deserves immediate further attention. In relation to our argument to balance interests, this appeal includes the suggestion to recognise the extraordinary immune competence of children and adolescents as far as SARS-CoV-2 infections are concerned, and to seriously scrutinise the use of compulsory vaccination for the young. If the concept of a ‘region of mild boosting’ is backed by additional evidence, for people having had previous encounters with the virus antigen (be it an encounter via SARS-CoV-2 itself or from a vaccination), then striving for early and regular re-infections might represent a viable alternative to avoid continuous re-vaccination – at least if no personal risk factors such as age or co-morbidity are present. The crucial point here is the idea that a re-infection for a previously infected person might be as harmless as a re-vaccination, be possibly of higher efficacy than a vaccination ^53^^,^^67^ as the immune system is interacting with the entire virus antigen, and finally, that such ‘mild boosting’ based on a re-infection could take place independently from the pharmaceutical industry and the high costs of universal vaccination (not to mention feasibility constraints). This last point is particularly important since many low resource countries are already struggling to implement first round vaccines and have limited budgets in which to afford continuous boosters. ^73^

In our eyes, an approach that factors natural immunity as a policy consideration should definitely be considered for persons without major comorbidities and those having ‘encountered’ the antigen in the past. Although the pursuit of this alternative will require further research evidence, we believe it could constitute a necessary and fundamental paradigm shift in coronavirus control, allowing us to ‘surf’ the waves instead of desperately trying to break them. An additional benefit of such a ‘surfing approach’ via a greater reliance on natural immunity is that such continuity of ‘antigen encounters’ will most probably lead – as Antia and Halloran point out – to the shift of the epidemic towards a milder childhood disease with limited long-term impact on health. This approach effectively could allow us to ‘live with the virus’ without continuous harmful NPIs nor the herculean task of boosting a global population of nearly 10 billion people on a continuous six-month rolling basis. ^76^ Most importantly, however, what this approach offers is a long-term alternative to current policies, which remain ad hoc, reactive, insufficient, inequitable, and devoid of any long-term exit strategy.

## Data availability

No data are associated with this article.
