# Supplementary material for: Surfing Corona waves – instead of breaking them: Rethinking the role of natural immunity in COVID-19 policy
Source: F1000Res. 2023 Aug 24;11:337. Originally published 2022 Mar 21. [Version 3] doi: 10.12688/f1000research.110593.3 (PMC10412939; doi:10.12688/f1000research.110593.3)
Supplement: Peer review history

# Peer review: Reviewer response for version 3

**DOI:** 10.5256/f1000research.154952.r199603

**Published:** 2023-08-29

**Authors:** Fareed Abdullah

**Affiliations:** 1 Division of Infectious Diseases, University of Pretoria, Pretoria, Gauteng, South Africa; 2 Department of Public Health Medicine, University of Pretoria, Pretoria, Gauteng, South Africa; 3 Office of AIDS and TB Research, South African Medical Research Council, Pretoria, South Africa

Approved

Is the topic of the opinion article discussed accurately in the context of the current literature?

Yes

Are arguments sufficiently supported by evidence from the published literature?

Partly

Are all factual statements correct and adequately supported by citations?

Yes

Are the conclusions drawn balanced and justified on the basis of the presented arguments?

Yes

Reviewer Expertise:

COVID-19, HIV

I confirm that I have read this submission and believe that I have an appropriate level of expertise to confirm that it is of an acceptable scientific standard.

# Peer review: Reviewer response for version 2

**DOI:** 10.5256/f1000research.139187.r188305

**Published:** 2023-08-09

**Authors:** Fareed Abdullah

**Affiliations:** 1 Division of Infectious Diseases, University of Pretoria, Pretoria, Gauteng, South Africa; 2 Department of Public Health Medicine, University of Pretoria, Pretoria, Gauteng, South Africa; 3 Office of AIDS and TB Research, South African Medical Research Council, Pretoria, South Africa

This opinion piece was submitted at a time when most countries and global institutions such as the WHO were still pondering the possibility of new surges arising out of new variants of concern. It argues that the place of non-pharmaceutical interventions such as lockdowns or mask mandates is very limited, given the high levels of natural immunity that has arisen out of the high prevalence of prior infection in all countries. It bemoans the fact that most governments at the time used the number of new cases diagnosed as the main risk measure against which to consider the re-introduction of non-pharmaceutical interventions. 

 The time that has elapsed since the opinion was submitted for publication has borne out the wisdom of the case they make. The article is still relevant as most countries in the world still consider the pandemic to be a threat and articulate public health policies recommending booster vaccines, whilst there is sufficient data to show that a single prior infection proffers significant protection against severe disease, as opined by these authors.

 The authors need to clarify that they are not criticising NPIs or vaccination campaigns at the beginning of the the pandemic but question their value after four pandemic waves.

 In the opinion piece the authors state, 'that vaccines alone cannot offer the miracle solution many governments had hoped for.' We suggest that the authors clarify that they have new infections in mind. Vaccines have proven to be less effective against infection but have certainly proven to be very effective against severe disease, ICU admission and death. 

 The statement that there has been 'slow progress of the coronavirus pandemic in most sub-Saharan African countries' is not based on evidenced as it is well known that the data from many of these countries and the testing capability has not been optimal. The evidence cited is from Canada. I suggest that the reference to sub-Saharan Africa be removed as it does not add any value to the arguments been posited.

 On vaccines (the 'fifth' consideration they suggest), the authors will do well to add a point about the how little the bivalent vaccines add to epidemic control given the additional cost of the bivalent vaccine. See Madhi *et al., *(2023) ^1^.

 Thank you for the opportunity to review this opinion piece.

Is the topic of the opinion article discussed accurately in the context of the current literature?

Yes

Are arguments sufficiently supported by evidence from the published literature?

Partly

Are all factual statements correct and adequately supported by citations?

Yes

Are the conclusions drawn balanced and justified on the basis of the presented arguments?

Yes

Reviewer Expertise:

COVID-19, HIV

I confirm that I have read this submission and believe that I have an appropriate level of expertise to confirm that it is of an acceptable scientific standard.

## References

[1] : Are bivalent vaccines better than ancestral-virus monovalent vaccines in protecting against severe omicron COVID-19?. *The Lancet Infectious Diseases* .2023; 10.1016/S1473-3099(23)00425-5 10.1016/S1473-3099(23)00425-5 37478878

# Peer review: Reviewer response for version 2

**DOI:** 10.5256/f1000research.139187.r152902

**Published:** 2022-10-31

**Authors:** Prof. Michael Marx

**Affiliations:** 1 Heidelberg Institute of Global Health, University of Heidelberg, Heidelberg, Germany

Having checked the revisions by the authors I do not have any objections to index the article now.

Is the topic of the opinion article discussed accurately in the context of the current literature?

Yes

Are arguments sufficiently supported by evidence from the published literature?

Partly

Are all factual statements correct and adequately supported by citations?

Yes

Are the conclusions drawn balanced and justified on the basis of the presented arguments?

Yes

Reviewer Expertise:

Health systems research; global health

I confirm that I have read this submission and believe that I have an appropriate level of expertise to confirm that it is of an acceptable scientific standard.

# Peer review: Reviewer response for version 1

**DOI:** 10.5256/f1000research.122215.r144183

**Published:** 2022-08-15

**Authors:** Prof. Michael Marx

**Affiliations:** 1 Heidelberg Institute of Global Health, University of Heidelberg, Heidelberg, Germany

This is an important paper that critically discusses the current COVID -19 policy on a global scale. A substantial part of the literature is being reflected to support the arguments.

 However, given the unprecedented speed and amount of research on SARS CoV 2 and the wealth of new literature that was produced since the article was submitted, some of the latest papers could be included, especially those dealing with immunological and virological features of new virus strains, e.g. Omikron BA.5,   and their public health impact.

 Moreover, although mentioned, more differentiation between northern and southern countries is missing especially when it comes to the impact of the pandemic related to incidence rates, demography, and respective immune competences and health care provision.   

 At some points, it is not clear enough which arguments are grounded on scientific evidence or which are based on assumptions deducted from current empirical knowledge. For instance, I would be more cautious to compare SARS CoV 2 with features of influenza virus strains.  

 It would add to the value of the paper if some statements would be a bit softened by admitting limitations due to still prevailing grey knowledge zones, e.g. immunity, future waves, and specific features of SARS CoV 2 virus strains. 

 Last but not least,  speaking of non-pharmaceutical intervention the value of preventive measures like wearing FFP2 masks, and Hepa- air filters shouldn't be underestimated when high incidence and morbidity rates risk compromising health care and social systems.

    

 However, with some updates and amendments, this article has the substance to further induce the critical and systemic discussion of the impact and control of the current pandemic.

Is the topic of the opinion article discussed accurately in the context of the current literature?

Yes

Are arguments sufficiently supported by evidence from the published literature?

Partly

Are all factual statements correct and adequately supported by citations?

Yes

Are the conclusions drawn balanced and justified on the basis of the presented arguments?

Yes

Reviewer Expertise:

Health systems research; global health

I confirm that I have read this submission and believe that I have an appropriate level of expertise to confirm that it is of an acceptable scientific standard, however I have significant reservations, as outlined above.

# Peer review: Reviewer response for version 1

**DOI:** 10.5256/f1000research.122215.r136136

**Published:** 2022-06-13

**Authors:** Cassian Minguet

**Affiliations:** 1 Centre académique de médecine générale, Université catholique de Louvain, Brussels, Belgium

This article asks some good questions. The question of natural immunity could be further referenced. The point of living with the virus (which is true) does not logically imply in my view that it necessarily means promoting natural immunity. On the other hand, concentrating vaccination on groups at risk (internationally) is indisputable. Similarly, taking into account the secondary effects (in terms of the general health of the population) of policies "breaking the waves" in order to make efficient and scalable decisions based on the advancement of knowledge is a major argument deserving to be even better highlighted in this article.

Is the topic of the opinion article discussed accurately in the context of the current literature?

Yes

Are arguments sufficiently supported by evidence from the published literature?

Partly

Are all factual statements correct and adequately supported by citations?

Yes

Are the conclusions drawn balanced and justified on the basis of the presented arguments?

Yes

Reviewer Expertise:

Family practice

I confirm that I have read this submission and believe that I have an appropriate level of expertise to confirm that it is of an acceptable scientific standard, however I have significant reservations, as outlined above.
